# *Streptococcus gordonii* finger infection: Case report and a review of the literature

**DOI:** 10.1097/MD.0000000000032506

**Published:** 2022-12-23

**Authors:** Cheng-Wei Kang, Xiao-Bing Pu, Gang Tan, Chang-Chao Dong, Zhao-Kui Yan, Li-Xue Wu

**Affiliations:** a Department of Orthopedics, West China School of Public Health and West China Fourth Hospital, Sichuan University, Chengdu, China; b Department of Pathology, West China School of Public Health and West China Fourth Hospital, Sichuan University, Chengdu, China.

**Keywords:** case report, duration of antibiotic, finger infection, plant infection, *Streptococcus gordonii*

## Abstract

Streptococcus gordonii is a rare cause of finger suppurative infection. Very few cases have been reported of its treatment.

**Patient concerns:**

A 68-year-old male of severe finger infection. Bacterial culture of synovial fluid revealed S gordonii.According to the patient’s history and auxiliary examination, the patient was diagnosed with S gordonii infection. Here, we review the diagnosis and treatment of this patient and describe the clinical and epidemiological characteristics of the patient.

**Diagnoses:**

Streptococcus gordonii finger infection.

Interventions: In the case of ineffective oral antibiotics, this patient chose to pursue an abscess incision, but in the course of treatment,the flexor digitorum tendon dissolved and eventually ruptured.

**Outcomes:**

The infection was controlled after intravenous injection of vancomycin. The incision was sutured 2 weeks later. No recurrence of infection was found after 3 months of follow-up.

**Lessons:**

The treatment included antibacterial and abscess treatments. In the absence of drug sensitivity results, antibiotics can be used empirically. If empirical anti-microbial treatment fails, the antibiotic regimen should be changed in a timely manner, Vancomycin may be an antibiotic choice

## 1. Introduction

*Streptococcus gordonii* is a subtype of *Streptococcus viridans*, and it is a natural inhabitant of the oral cavity.^[1]^ It is usually related to the formation of dental plaques. *S gordonii* is a gram-positive, nonmotile, and facultative anaerobic bacterium. It has been confirmed that *S gordonii* can colonize the heart valve and is the main pathogen of bacterial endocarditis.^[2]^ *Staphylococcus aureus* is the most common microorganism in adult joint infections, accounting for 40%‐60% of the cases,^[3]^ followed by β-hemolytic Streptococcus, which is also a common pathogenic bacteria causing joint infections. In contrast, comprehensive reviews regarding joint infections do not mention cases of joint infection caused by the *S viridans* group.^[4]^ There are currently no reported cases of finger soft tissue infections caused by *S gordonii*.^[5]^ We describe a case of finger infection caused by the direct inoculation of *S gordonii*.

## 2. Case presentation

**Figure 1.**
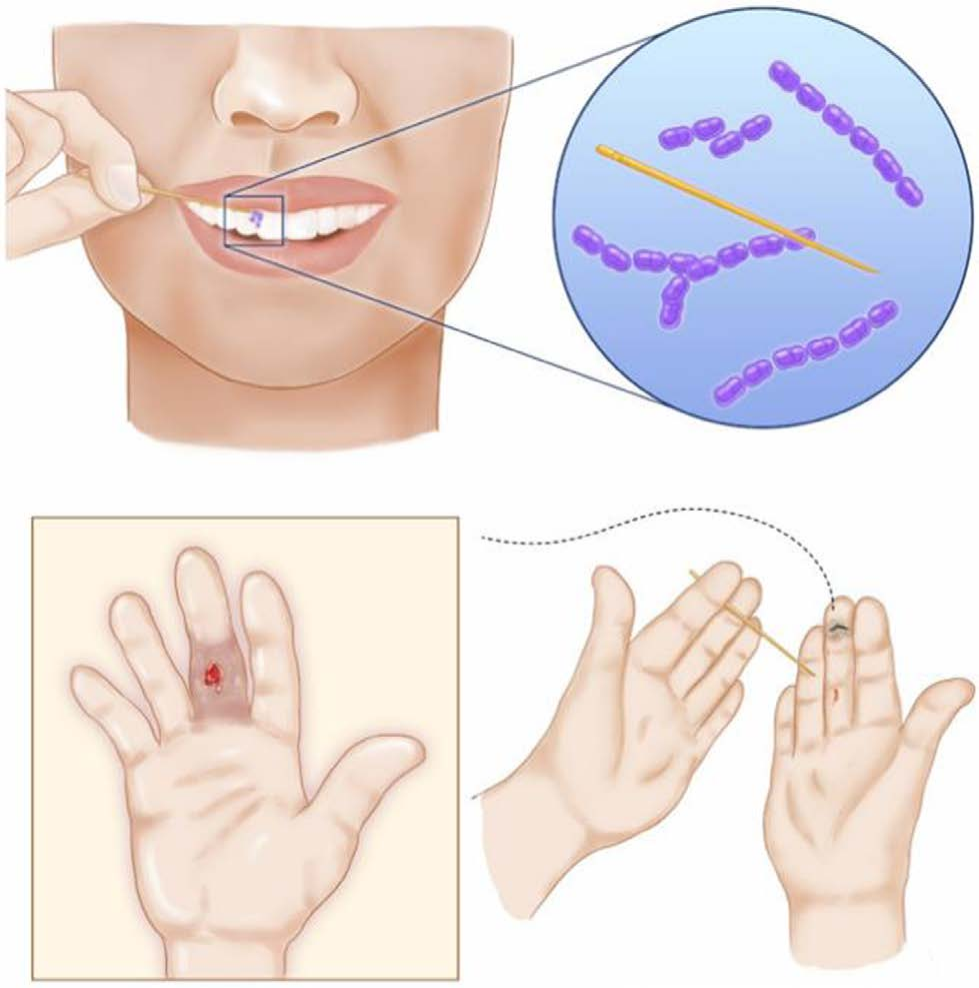
Schematic diagram of infection caused by a toothpick stabbing the right middle finger.

A 68-year-old Caucasian male was admitted to the hospital for over 1 month because the middle finger of his right hand was previously stabbed by a sharp instrument, resulting in swelling, pain and purulent exudate. Past medical history revealed type 2 diabetes and hyperthyroidism. This patient had the habit of using a toothpick to pick his teeth after meals. One month before admission, the patient used his left hand to hold a toothpick to pick his teeth after eating. During the process of picking his teeth, he found that a mosquito wanted to land on his right hand. The patient patted the mosquito with his left hand without discarding the toothpick. During the process of patting, the palm of the middle finger of his right hand was accidentally stabbed by the toothpick, and he immediately pulled the toothpick out of his hand without consulting a healthcare provider. Three days later, the soft tissue of the injured right middle finger exhibited swelling, pain, and exudate, so the patient went to the local community clinic. The clinic issued “cefaclor,” an oral antibiotic treatment for 1 week. The symptoms of the affected finger did not improve and were getting worse. The patient went to another large orthopedic hospital and was diagnosed with a right middle finger infection. The outpatient department lanced and drained the right middle finger under local anesthesia. After 5 days of undergoing dressing changes in the outpatient wound treatment room, the pain and swelling in the patient’s finger were still not significantly improved. The patient went to the outpatient orthopedic department and had dressing changes applied in the wound outpatient department of our hospital, but the response to treatment was still poor. The patient went to Wuhou District People’s Hospital of Chengdu City for further treatment. The outpatient department performed a right middle finger wound expansion incision and did dressing changes once a day for 1 week. The patient’s finger was rinsed with normal saline, iodophor solution, and hydrogen peroxide during each dressing change. After 1 week, the wound infection was still not controlled (Fig. 1).

For further treatment, the patient went to the orthopedic clinic of our hospital and was diagnosed with a right middle finger soft tissue infection, so he was hospitalized. Physical examination revealed that the patient had no chills or fever, and his temperature, heart rate, and cardiac auscultation were normal. Local physical examination: Vision: The proximal part of the middle finger of the right hand had an obvious odor, and the tension was relatively high. There were swelling and purulent secretions seen in the 1 cm longitudinal incision made. There was flexor tendon exposure, lysis, and necrosis; touch: the temperature of the skin of the middle finger of the right hand was increased, accompanied by tenderness, and the skin sensations on both sides of the right hand were not significantly decreased; Smell: The wound secretion was malodorous. Momentum: the range of motion of the metacarpophalangeal joint of the proximal middle finger of the right hand was limited. Imaging examination: Radiography of the right hand demonstrated no obvious abnormalities.

**Figure 2.**
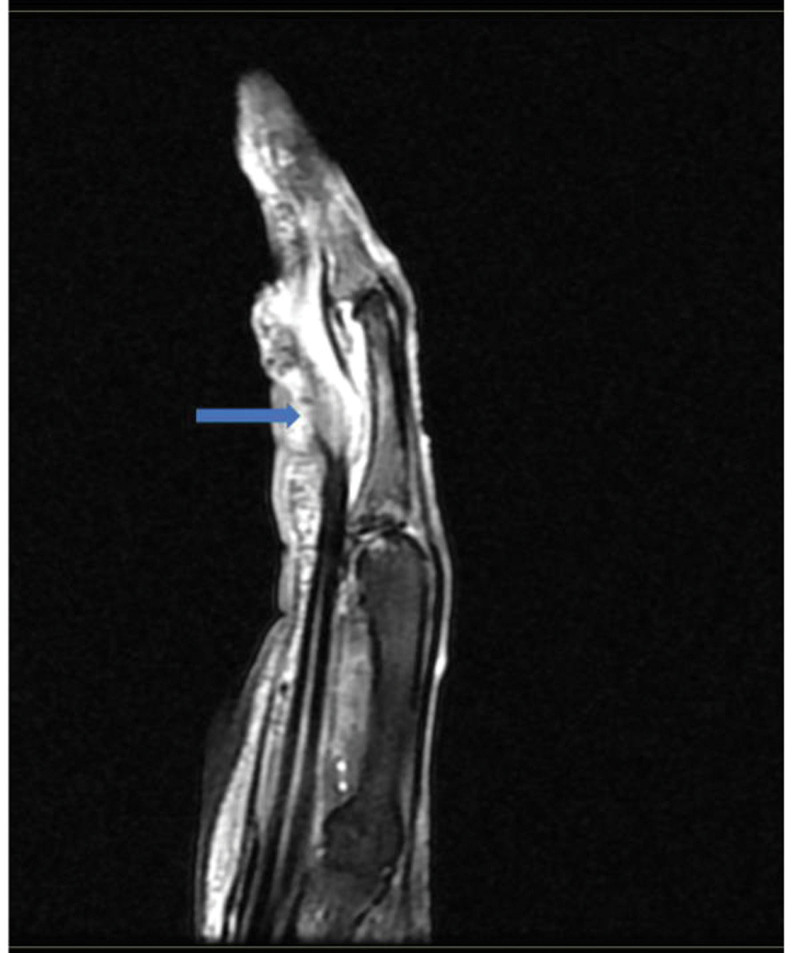
Sagittal magnetic resonance imaging (MRI) of the right middle finger. The blue arrow indicates the rupture of the flexor tendon.

Laboratory investigation: white blood cell and neutrophil count, C-reactive protein (CRP), and procalcitonin were normal, and blood cultures were negative. Fasting blood glucose was 6.77 mmol/L↑; glycosylated hemoglobin was 6.7%↑; and erythrocyte sedimentation rate (ESR) was 33 mm/h↑. Aspiration of the finger revealed a purulent exudate with a WBC count of 31,500 cells/mm^3^. The microorganism that was observed on a Gram-stain of the synovial fluid was a gram-positive cocci+ (single, double, mass); the organism was identified by mass spectrometry (matrix-assisted laser desorption/ionization-time of flight mass spectrometry, MALDI-TOF) as *S gordonii*. Pure culture of the alpha-hemolytic streptococci was obtained after 24 h of incubation of the synovial fluid inoculated into aerobic BACTEC system. An MRI of the right hand revealed signal continuity interruption in the flexor tendon of the right middle finger (Fig. 2).

### 2.1. Treatment, outcome and follow-up

**Figure 3:**
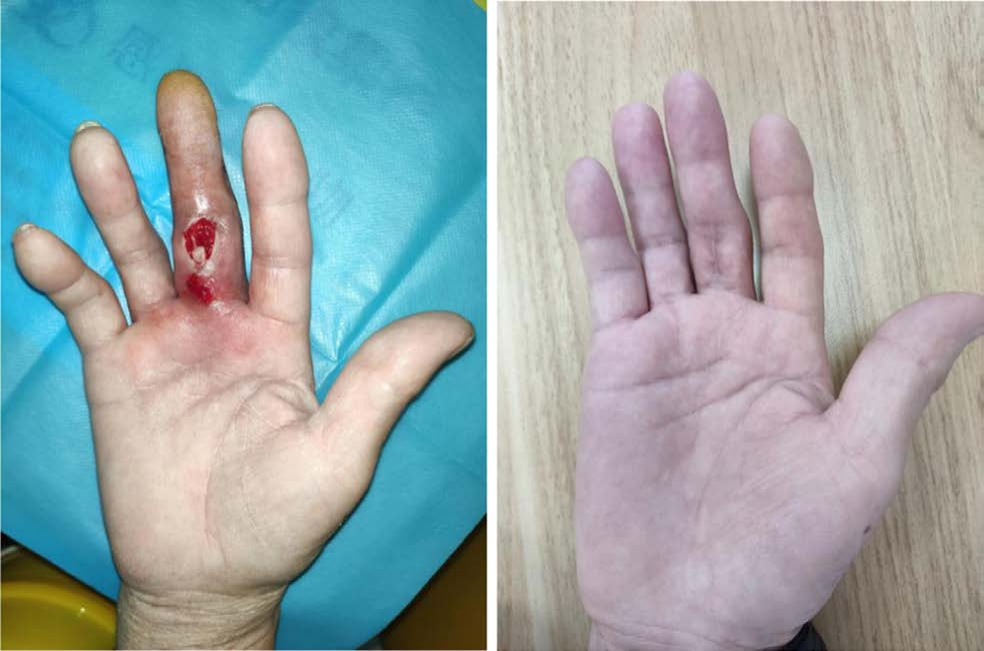
(A and B) The appearance of swelling and exudation of the finger when the patient was admitted to our hospital; Follow up the appearance of fingers 3 months after discharge.

Treatment: a consultation in the Infectious Disease was obtained for guidance regarding antibiotic therapy. The patient was started on intravenous (IV) first try piperacillin-tazobactam (4.5 g/8 hourly) combined with penicillin G (3.2 million units/6 hourly) based on the consultation recommendations. After 1 week of treatment, the symptoms of redness, swelling, pain, and purulent secretion of the affected finger were still not significantly improved. After consultation with the Infectious Disease Department again, the antibiotic was adjusted to vancomycin (1 g/12 hourly). After vancomycin was given intravenously for 2 weeks, the swelling of the right middle finger subsided, the pain was significantly reduced, the flexion activity was still limited, and there was no numbness, fever, or shivering. Then, the patient was switched to oral levofloxacin 500 mg od/day for 2 weeks. Physical examination at that time revealed that the swelling of right middle finger subsided, and there was no purulent exudate. The skin temperature of the right middle finger was normal, tenderness was mild, and skin sensation on both sides of the right hand were not significantly decreased. Momentum: The movement function of proximal metacarpophalangeal joint of the right middle finger was partially limited. We collected synovial fluid twice for bacterial culture, which were both negative. The patient declined the reconstruction of the flexor digitorum tendon. Considering that the infection was under control, the wound was sutured, and the patient was discharged. The suture was removed 2 weeks after discharge, and the wound healed well after 3 months of follow-up (Fig. 3A and B).

## 3. Discussion

**Figure 4.**
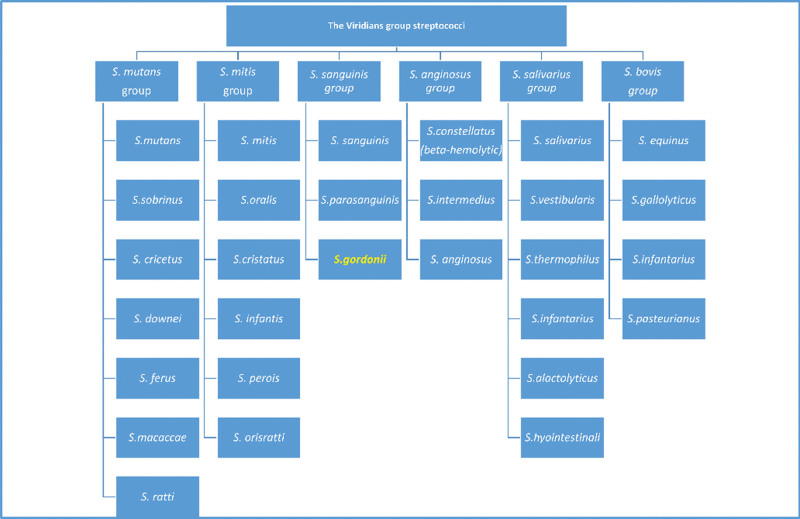
Major subclassified of the *Streptococcus viridans* group.

*S gordonii* is a gram-positive, nonmotile, coccal, facultative anaerobic genus of the viridans group Streptococcus.^[6]^ The Streptococcus viridans group is a heterogeneous group of organisms with controversial taxonomy^[7]^ and can be further subclassified into 6 major groups: the *Streptococcus anginosus* group, *Streptococcus mitis* group, *Streptococcus sanguinis* group, *Streptococcus bovis* group, *Streptococcus salivarius* group, and *Streptococcus mutans* group. The *S sanguis* group includes *S sanguis*, *Streptococcus parahaemolyticus*, and *S gordonii*^[8]^ (Fig. 4).

The viridans group streptococci are part of symbiotic flora with low toxicity. Infection with this microorganism usually occurs at the site of a previous injury.^[9]^ In some reported isolated cases, *S viridans* mainly affects the knee, sternoclavicular, acromioclavicular, and sacroiliac joints.^[10–12]^ Alpha-hemolytic streptococcus has been associated with 15 cases of spondylitis, including infections with the *S anginosus* and *S mitis* groups (*S mitis* and *s. sanguinis*).^[13]^ Other members of Streptococcus viridans from the *S mitis* group (*S sanguinis* and *S oralis*) have been reported in cases of septic arthritis.^[14–16]^ Hafsa Farooq et al reported a case of empyema caused by *S gordonii* infection.^[17]^ Ziv Dadon et al reported 2 cases of spondylitis caused by *S gordonii* infections.^[18]^ However, we have not found any cases of *S gordonii* finger infections in the literature.

*S gordonii* is a kind of oral symbiotic bacteria that has no direct pathogenicity; it is related to dental plaque formation and endocarditis by entering the bloodstream, generally secondary to oral trauma.^[19,20]^ It can spread from mouth and cause systemic infection.^[6]^ Oral mucositis is a confirmed risk factor.^[21]^ Similar to *S sanguis* and *S oralis*, *S gordonii* is often found in the blood culture of patients with infective endocarditis.^[22]^ *S gordonii* colonizes platelet-fibrin thrombi on abnormal heart valves or the endocardium, leading to heart valve damage and dysfunction.^[7,23]^ In addition, cases of spontaneous bacterial peritonitis and subcutaneous abscesses can be caused by *S gordonii*.^[24,25]^ Furthermore, septic knee arthritis and periprosthetic infection after joint replacement caused by *S gordonii* have been reported.^[26–28]^ The route of infection in these cases is presumed to be transient bacteremia.^[26]^ Surface-associated adhesins are thought to play an important role in the pathogenicity, and genes encoding several of these proteins, such as collagen adhesion protein (CNA), cell wall-associated protein (WapA), Streptococcus surface protein A (SspA), C5a peptidase (Scp), IgA1 protease and enolase, have been identified as associated factors.^[29]^

Gram-negative bacteria are more common in elderly patients and immunocompromised patients than in young people. Anaerobes rarely cause septic arthritis, but they are more likely to occur when there is a history of penetrating trauma.^[5]^ However, an extensive review of the literature shows that this is the first case of hand infection caused by the direct inoculation of *S gordonii.*

The key method for the rapid and reliable diagnosis of suspected suppurative arthritis is the microbiological examination of synovial fluid. It is best to smear and culture the exudate before using antibiotics, and the aspirated exudate should be sent to the laboratory immediately for testing. Inoculating synovial fluid into blood culture medium, such as the Becton-Dickinson diagnostic instrument system or pediatric isolator tubes, can improve the recovery and identification rates of microorganisms. Matrix-associated laser desorption ionization-time of flight (MALDI-TOF) is a rapid and cost-effective method for the identification of bacterial cultures. Recent studies have compared the capabilities of this system with biochemical analysis, suggesting that MALDI-TOF seems to be a rapid and accurate technique for the identification of viridans group streptococci.

The main treatment for abscesses includes antibiotics and exudate removal. Purulent material can be removed by surgery or closed needle aspiration. Whether an invasive operation is needed and which method should be used to remove pus remain controversial.^[30,31]^ The specific treatment depends on the time of infection, the type of organism involved, and the specific condition of the patient’s wound, which are judged by the experience of the doctor. The early identification of microorganisms and the timely use of appropriate antibiotics may help to avoid surgical treatment.

The finger of this patient was infected after being stabbed with a toothpick. The microorganism cultured from the wound exudate was identified as *S gordonii*, which is a symbiotic bacterium in the oral cavity. Based on to the patient’s injury, the route of infection of the finger was confirmed. When the patient was infected, the community clinic prescribed “cefaclor” antibiotic treatment as invalid. According to the cause of the disease, this case suggests that outpatient doctors should think of the possibility of oral symbiotic bacterial causing infection. Currently, the choice of antibiotics is based on the organism involved, the results of Gram staining and the culture results. If an abscess has formed, the purulent material can be aspirated for bacteriological analysis. If laboratory tests are available, the drug sensitivity tests should be further evaluated. The patient went to an orthopedic hospital for abscess lancing and drainage after an ineffective oral antibiotic treatment for 1 week and the aggravation of soft tissue infection. This kind of treatment can be considered. However, it was found during dressing changes in the outpatient department that the patient’s flexor tendon of the affected finger was dissolved and ruptured. It is speculated that this is related to the repeated use of hydrogen peroxide for flushing. We recommend that a large amount of normal saline should be used alone for flushing these types of wounds. The continued use of hydrogen peroxide may lead to granulation tissue damage and serious tendon lysis.

The patient received intravenous antibiotics in our hospital to treat the infection. He first received piperacillin-tazobactam combined with a penicillin G intravenous drip for antibacterial treatment. After 5 days of treatment, the symptoms of swelling, pain, and discharge in the patient’s finger did not improve. We adjusted the antibiotic regimen in time and used vancomycin for antibacterial treatment. The patient experienced relief after 3 days, and the swelling and pain of the finger were relieved. After 2 weeks, the appearance of the wound returned to normal. An extensive literature search did not find any reports of hand infection caused by *S gordonii*, and there was no information in the literature about the process of infection and how long antibiotic treatment might take. We twice sampled synovial fluid from the wound for further bacterial culture, both of which were negative. The wound was eventually sutured after confirming the control of the infection. The sutures were removed 2 weeks after the operation, and the wound healed well after 3 months of follow-up.

## 4. Conclusions

*S gordonii* is a gram-positive, nonmotile, facultative anaerobic bacterium that is most common in the oral cavity. This case represents the first known case of septic arthritis of the hand with direct infection of *S gordonii*. Treatment included antibacterial and abscess drainage treatment, but the treatment is still controversial. If the empirical use of antibiotics is not effective, the antibiotic regimen should be changed in a timely manner. At present, there is no exact guide for the duration of antibiotic use. After the infection control of fingers is judged by the appearance, the wound can be sutured if the bacterial culture of secretions is negative.

## Acknowledgments

The authors wish to thank Jing Zhang’s microbiological management and laboratory confirmation, Li-bo Yan’s antibiotic guidance and Dongxin Huang’s illustration for this article.

## Author contributions

KCW: clinical management of the cases, wrote the paper; PXB: lecture of the manuscript, initiated the project; WLX supplementary retrieval of patient data and review of case reports, critical review of the manuscript, overall responsibility for the manuscript; DCC, TG and YZK: orthopedic care of the patient. All authors read and approved the final manuscript.

**Conceptualization:** Xiao-bing Pu.

**Data curation:** Chang-chao Dong, Gang Tan, Zhao-kui Yan.

**Project administration:** Li-xue Wu.

**Writing – original draft:** Chengwei Kang.
